# Modification of N-glycosylation sites allows secretion of bacterial chondroitinase ABC from mammalian cells

**DOI:** 10.1016/j.jbiotec.2009.11.002

**Published:** 2010-01-15

**Authors:** Elizabeth M. Muir, Ian Fyfe, Sonya Gardiner, Li Li, Philippa Warren, James W. Fawcett, Roger J. Keynes, John H. Rogers

**Affiliations:** a Department of Physiology Development and Neuroscience, University of Cambridge, Downing St., Cambridge CB2 3EG, UK; b Cambridge Centre for Brain Repair, University of Cambridge, Forvie Site, Robinson Way, Cambridge CB2 2PY, UK

**Keywords:** Glycosylation, Protein secretion, Endoplasmic reticulum, Chondroitinase, Spinal cord injury

## Abstract

Although many eukaryotic proteins have been secreted by transfected bacterial cells, little is known about how a bacterial protein is treated as it passes through the secretory pathway when expressed in a eukaryotic cell. The eukaryotic N-glycosylation system could interfere with folding and secretion of prokaryotic proteins whose sequence has not been adapted for glycosylation in structurally appropriate locations. Here we show that such interference does indeed occur for chondroitinase ABC from the bacterium *Proteus vulgaris*, and can be overcome by eliminating potential N-glycosylation sites. Chondroitinase ABC was heavily glycosylated when expressed in mammalian cells or in a mammalian translation system, and this process prevented secretion of functional enzyme. Directed mutagenesis of selected N-glycosylation sites allowed efficient secretion of active chondroitinase. As these proteoglycans are known to inhibit regeneration of axons in the mammalian central nervous system, the modified chondroitinase gene is a potential tool for gene therapy to promote neural regeneration, ultimately in human spinal cord injury.

## 1 Introduction

In several situations there is therapeutic potential in directed secretion of bacterial proteins from eukaryotic cells. Examples are the expression of bacterial epitopes for vaccination against bacterial pathogens (Lowrie et al., 1999), the characterisation of bacterial proteins that stimulate T cells (Wilson et al., 2000), and expression of bacterial inhibitors of cell proliferation as a potential treatment for cancer (de la Cueva-Mendez et al., 2003). However, secretion of such proteins may be compromised by the presence of cryptic signals in the bacterial sequence that cause inappropriate modifications in animal cells, such as N-glycosylation.

In mammalian cells, almost all secreted proteins contain Asn-X-(Ser/Thr) motifs (N-X-S/T) which are N-glycosylated co-translationally in the endoplasmic reticulum, to assist and validate the correct folding of the protein (Gahmberg and Tolvanen, 1996; Dempski and Imperiali, 2002). Although an eukaryote-like N-glycosylation system exists in the bacterium *Campylobacter jejuni* (Szymanski and Wren, 2005), most bacteria do not perform this operation. Therefore, genes from most bacterial species may encode N-X-S/T at positions where, if expressed in mammalian cells, they will be N-glycosylated. Such modifications could interfere with enzyme folding, impeding transit through the secretory pathway, and/or cause loss of activity by alteration of the active site or by sterically hindering substrate binding. Misfolding of a protein could also direct it to the proteosome for degradation. It is therefore likely that efficient secretion of bacterial proteins from mammalian cells may require selective mutagenesis of potential N-glycosylation sites if they are present.

We aim to transduce mammalian cells to secrete the bacterial enzyme chondroitinase ABC, with a view to gene therapy to promote regeneration of neural pathways in the human central nervous system (CNS), particularly in spinal cord injury. Spinal cord injuries leave large numbers of people permanently paralysed every year, as the damaged axons fail to regenerate. Injury causes a glial ‘scar’ to form in the spinal cord, and cells in this scar zone express molecules that are inhibitory to axon regrowth (Silver and Miller, 2004; Yiu and He, 2006; Kwok et al., 2008). Together with growth-inhibitory proteins released by degenerating myelin, these molecules prevent regenerative axon growth and functional recovery. Measures designed to stimulate regeneration have had significant success in rodent models (Silver and Miller, 2004; Ramer et al., 2005; Thuret et al., 2006; Bradbury and McMahon, 2006; Kwok et al., 2008).

A significant contribution to axon growth inhibition is made by chondroitin sulphate proteoglycans (CSPGs) (Kwok et al., 2008). These molecules, such as neurocan and NG2 (Dou and Levine, 1994; Levine and Nishiyama, 1996; Bovolenta and Fernaud-Espinosa, 2000; Silver and Miller, 2004; Tan et al., 2006; Yiu and He, 2006), have long sulphated glycosaminoglycan (GAG) chains attached to their core protein component, and the GAG chains are responsible for much of the inhibitory activity (Grimpe and Silver, 2004).

Chondroitinase ABC is a bacterial enzyme that degrades these inhibitory carbohydrate chains. It is active when injected into the mammalian CNS, as it depletes GAG immunoreactivity surrounding an injury site and concomitantly generates carbohydrate products which are not seen in normal tissue (Moon et al., 2001; Bradbury et al., 2002; Kwok et al., 2008). The use of chondroitinase promotes axon regeneration in rats, as demonstrated first in the nigrostriatal tract (Moon et al., 2001) and also in the spinal cord (Bradbury et al., 2002). Repeated injections of chondroitinase at the site of spinal cord injury promoted extensive regeneration of both corticospinal and sensory axons, accompanied by significant functional motor recovery (Bradbury et al., 2002). These results have been confirmed and extended by others (Kwok et al., 2008). Chondroitinase also enhances axon regeneration in combination with grafts of Schwann cells and/or olfactory ensheathing cells or neural stem/progenitor cells (reviewed by Kwok et al., 2008).

As well as removing the block to axon regeneration by CSPGs, chondroitinase may promote recovery by other mechanisms, including dispersal of other axon-inhibitory molecules, neuroprotection (Carter et al., 2008), and sprouting and synaptic plasticity in spared pathways due to removal of perineuronal nets (Crespo et al., 2007; Galtrey et al., 2007). Whatever the predominant mechanism, chondroitinase is clearly a promising treatment for spinal cord injury.

However, the use of chondroitinase in human CNS will require modification. Delivery of the enzyme by local injection into the spinal cord region is technically problematic, for several reasons. Chronic infusion or repeated injections would be required to relieve axon growth inhibition for the extended periods needed for functional recovery. Chronic delivery carries risks of tissue damage, infection, and immunogenicity. A solution to these problems would be to transfect neurons and/or glia at the injury site with a vector containing the gene for chondroitinase, so they secrete the enzyme themselves.

Our strategy, therefore, is to modify the bacterial chondroitinase ABC sequence so that mammalian cells synthesise and secrete active enzyme. In this study, we show that N-glycosylation is indeed an obstacle to secretion of chondroitinase from transfected mammalian cells, and that it can be overcome by selective mutation of key N-glycosylation sites.

## 2 Materials and methods

### 2.1 Initial sequence

The sequence of the gene for *Proteus vulgaris* chondroitinase ABC was reported by Ryan et al. (1994) (Entrez accession number AAB43331 = gi1828877) and confirmed by Prabhakar et al. (2005a). The encoded sequence was also confirmed from the protein crystal structure by Huang et al. (2003). These studies showed that an independent sequence reported by Sato et al. (1994) contained several errors.

We obtained a cDNA for *P. vulgaris* chondroitinase ABC in a prokaryotic expression vector, although the coding region lacked an N-terminal signal sequence to direct secretion from cells, and had two inactivating mutations. Apart from these changes, the clone encoded the same sequence reported by Ryan et al. (1994). We corrected the two mutations by site-directed mutagenesis, and added a eukaryotic signal sequence from mouse matrix metalloprotease 2 (GenBank accession no. NM008610 = gi47271505) (Reponen et al., 1992). An optimised Kozak sequence was also inserted at the 5′ end to allow recognition by eukaryotic ribosomes and to maximise protein yield. We further changed some of the codons that are unfavourable for translation by mammalian ribosomes, replacing them with those used more frequently by mammalian cells. The resulting ‘initial sequence’ is shown in Supplementary Fig. S1.

This modified cDNA was subcloned into the eukaryotic expression vector pcDNA 3.1 (Invitrogen), in which transcription is directed by the cytomegalovirus promoter, which directs high-level expression in a wide range of mammalian cells. This initial clone is named C4.

Protein structure was visualised using RasMol Molecular Graphics Visualisation Tool (by Roger Sayle: http://www.rasmol.org).

### 2.2 Site-directed mutagenesis

Mutagenesis was carried out using the QuikChange Multi-Site-Directed Mutagenesis kit (Stratagene). All mutagenesis was carried out using constructs inserted in pcDNA 3.1.

Primers containing the desired mutations (Supplementary Fig. 2) were designed where possible to insert or delete a restriction site to allow easy identification of mutant clones. The primers were modified with a 5′ phosphate and PAGE-purified to improve mutation efficiency. All clones were sequenced to confirm successful mutagenesis. Each construct was assayed using the TNT system (below) to assess the effect of the mutation on enzyme activity. This allows us to distinguish the direct effect of the mutation on enzyme activity separately from any effects due to glycosylation inside the cell.

### 2.3 *In vitro* transcription–translation

*In vitro* transcription/translation (IVTT) reactions were carried out with rabbit reticulocyte lysate in a coupled reaction with T7 polymerase, using the TNT Quick Coupled Transcription/Translation kit (Promega). Each 25 μl reaction contained 1 μg of plasmid. Labeled reactions also included 1 μl biotinylated lysine (Transcend) or 1 μl ^35^S-methionine (Redivue l-methionine, 37 MBq/mmol, Amersham). Reactions to assess glycosylation also contained 1 μl of canine microsomes (Promega). The samples were incubated at 30 °C for 90 min. The products of the IVTT reactions were then separated by sodium dodecyl sulphate polyacrylamide gel electrophoresis (SDS-PAGE), as follows:

*Western blots for chondroitinase ABC*: 5 μl samples were run on 6% reducing gels and blotted as described below.

^35^*S-labeled samples*: 5 μl samples were run on 8% Tris/glycine gels (Invitrogen), fixed in methanol/acetic acid, and incubated in Amplify (Amersham) for 30 min prior to drying. The dried gels were then exposed to X-ray film for 2 h.

*Biotin-labeled samples*: 1–2 μl samples of IVTT reactions labeled with biotinylated lysine were run on 10% Tris/glycine gels, transferred to nitrocellulose membrane using a semi-dry-blot (Invitrogen), and then probed with strepavidin-linked horse radish peroxidase (Promega), prior to development using chemiluminescence (Promega). All chemiluminescence products were detected using chemiluminescence film (Amersham).

*Protease protection assay* (Schmidt-Rose and Jentsch, 1997): The translation mixture was brought to 10 mM CaCl_2_ and chilled on ice. Aliquots of 10 μl were incubated with Proteinase K, 30 μg/ml (Roche), in the presence or absence of 1% Triton X-100. Controls remained without proteinase and detergent. Proteolysis proceeded on ice for 60 min and was stopped by adding 5 mM phenylmethylsulphonyl fluoride. After 10 min on ice, 50 μl of preheated sample buffer (95 °C) was added and the sample was boiled for 15 min to inactivate the protease. The samples were then run on a gel.

### 2.4 Morgan–Elson reaction

This reaction measures chondroitinase activity by the N-acetylation of product disaccharides and subsequent reaction to give a coloured product (Morgan and Elson, 1934; Reissig et al., 1955). The procedure was adapted from one given at <http://www.acciusa.com/seikagaku/products/product.asp>.

Initially the reaction resulted in unstable colour intensity due to the formation of precipitate during the reaction, as previously noted (Takahashi et al., 2003). Therefore, activity could only be estimated qualitatively. We then refined the procedure by centrifuging at two stages to minimise the presence of precipitate at the end, thereby allowing spectrophotometry to be used to quantify activity.

The reaction mixture was made up of 100 μl of 40 mM sodium acetate, 40 mM Tris–Cl pH 8.0, 10 mg/ml chondroitin-6-sulphate (Sigma), mixed with 20 μl enzyme sample (IVTT product or standard). *P. vulgaris* chondroitinase ABC (Sigma) was used as standard. The reaction was incubated at 37 °C for 20 min, then stopped by boiling for 1 min. Potassium borate solution (0.8 M, pH 9.1, 100 μl) was added and the mixture was boiled for 7 min. It was chilled on ice then centrifuged in a microfuge at 13,000 rpm for 10 min. To the supernatant, 1 ml glacial acetic acid was added and mixed before centrifugation for a further 20–30 min. To 1 ml of supernatant, 0.4 ml of Morgan–Elson Reagent (10 g para-dimethylamino-benzaldehyde in 100 ml in glacial acetic acid with 12.5% concentrated HCl) was added and incubated at 37 °C for 20 min. Product was measured by absorbance at 550 nm. (This wavelength gives higher and more consistent absorption values than the standard 585 nm.)

### 2.5 Cell culture and transfection

Neu7 cells (Smith-Thomas et al., 1994; Fok-Seang et al., 1995) were grown in Dulbecco's modified Eagle's medium (DMEM) containing 10% fetal bovine serum and 10% horse serum. When cells had just reached confluence, the medium was replaced by DMEM with ITS^3+^, for 48 h, providing conditioned medium for use on transfected cells.

COS7 cells were grown in Optimem (Gibco-Invitrogen) with 10% fetal bovine serum. Media for all cell cultures were supplemented with standard concentrations of penicillin, streptomycin and fungazone.

Primary glial cells were cultured from cortex of Wistar rat pups 1–2 days old (Muir et al., 2002), grown in DMEM with 10% fetal bovine serum, passaged after about one week (leaving a culture consisting mainly of astrocytes), and used for transfection several days later.

Transfection was performed in 25-cm^2^ flasks with the cells ∼60–70% confluent, with polyethyleneimine (PEI) or FuGene, and pAdVAntage vector to increase translation efficiency (Promega), using standard procedures. After 24 h, the medium was replaced with Neu7 conditioned medium. This conditioned medium was collected after 24 h, centrifuged to remove detached cells, and concentrated 5–10-fold by centrifugation in a Centricon-50 unit (Millipore), mixed with protease inhibitor cocktail (Sigma P8340), and frozen for subsequent electrophoresis.

In every round of transfections, one transfection was performed with GFP to assess transfection efficiency by fluorescence microscopy of the live cells, and in some cases by fixation and counterstaining with bisbenzamide (Hoechst 33258). Transfection efficiencies were 17–50% for COS cells.

### 2.6 Western blots

Sodium dodecyl sulphate polyacrylamide gel electrophoresis (SDS-PAGE) and Western blotting were done by standard techniques. For detection of proteoglycans, samples of concentrated conditioned medium were either 50 μl or the volume containing 200 μg protein. Controls were similar samples from non-transfected cells, one of which was digested with chondroitinase ABC (Sigma, 20 mU) at 37° for 3 h. Samples were mixed with 10 μl of 5× non-reducing Laemmli sample buffer, boiled for 2 min, separated by SDS-PAGE (5% acrylamide gel), and electroblotted in a Transblot Semi-dry Transfer Cell blotter (Bio-Rad) to Hybond-ECL membrane.

IVTT product samples consisted of 5 μl IVTT product, 6 μl 5× reducing Laemmli sample buffer, 19 μl chondroitinase buffer (40 mM NaAc, 40 mM Tris–Cl pH 8.0). Proteins were separated by SDS-PAGE (6% acrylamide gel) and transferred to Hybond-ECL membrane.

Antibodies were: mouse anti-NG2 (Santa Cruz sc33666 = mcAb 132.38), diluted 1:1000; mouse anti-‘stub’ (Seikagaku, mcAb 1B5, 1:250); rabbit anti-chondroitinase ABC (Acorda Inc., 1:2000, pre-absorbed by incubation with conditioned medium from Neu7 cells for 3 h at room temperature). The anti-‘stub’ antibody detects an epitope revealed by chondroitinase action, remaining attached to the CSPG core proteins as a so-called ‘stub’ after chondroitinase ABC has cleaved off most of the CSPG chains. The CSPG repeating disaccharide unit, d-glucuronic acid linked to N-acetyl-galactosamine (variously sulphated), is masked in native CSPG, but is revealed after chondroitinase digestion. Chondroitinase ABC is largely specific for sulphated units, and antibody 1B5 (Couchman et al., 1984) recognises the unsulphated unit (Δ-di-0S) that remains behind, thus acting as a sensitive probe for chondroitinase activity.

Membranes were incubated in 2% ECL Advanced Blocking Agent (Amersham) in Tris-buffered saline with 0.1% Tween-20 (TBS-T) at room temperature for 3–4 h, then incubated with primary antibody in blocking solution, overnight at 4 °C. Membranes were washed in TBS-T before incubation with secondary antibody (peroxidase-labeled anti-mouse or anti-rabbit IgG, 1:10,000 to 1:30,000 in blocking solution) for 1 h at room temperature. Membranes were washed in TBS-T before reaction with ECL chemiluminescence detection reagent and visualisation on Hyperfilm (Amersham).

## 3 Results

### 3.1 Recombinant chondroitinase ABC is active *in vitro*

We constructed a cDNA clone that encodes the natural (bacterial) sequence of chondroitinase ABC, with a Kozak sequence to initiate translation in mammalian cells, and the mouse MMP2 signal sequence to direct secretion of the enzyme, and some codons modified to be more favourable for mammalian expression (sequence in Supplementary Fig. S1; see Section 2). This ‘initial’ cDNA, subcloned into the expression vector pcDNA 3.1, was named clone C4.

This vector was sufficient for active enzyme to be produced when testing the construct in an *in vitro* transcription/translation (IVTT) system using rabbit reticulocyte lysate, followed by protein gel electrophoresis. A protein of the expected size was synthesised; it was immunoreactive with antibody against chondroitinase ABC; and active enzyme could be detected by colorimetric assay. However, no secreted enzyme activity was detected after transfection of COS7 cells with the construct, even though RT-PCR confirmed that the cDNA was efficiently transcribed by the cells (data not shown).

One explanation for the lack of detectable secreted product may be the presence of cryptic signals in the bacterial protein which are inappropriately recognised and processed by eukaryotic cells. Computer prediction programs established the absence of the endoplasmic reticulum retention signal KDEL and signal sequences directing proteins to organelles, but we did find 17 predicted sites for N-glycosylation in the protein sequence. Such modifications could interfere with enzyme folding and/or transit through the secretory pathway.

**Fig. 1:**
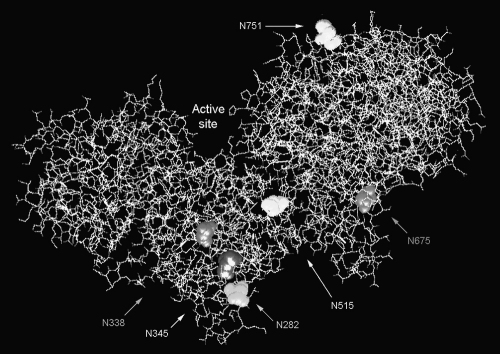
3D structure of bacterial chondroitinase ABC, showing putative glycosylation sites that have been eliminated. The structure is from Huang et al. (2003). The active site is on the right-hand flank of the cleft (Prabhakar et al., 2005b). Six potential N-glycosylation sites predicted to affect enzyme structure or activity are highlighted. Different constructs differ as follows (see Table 1): B5 v C4, Asn 751; B1 v B5, Asn 515; X12 v B1, Asn 345; X30 v B1, Asn 282; and Y13 v A10, Asn 338. (A seventh site, Asn-836, was mutated in one clone but is not glycosylated.) See Supplementary Fig. S3 for coloured version.

A protein 3D-imaging program was used to locate the potential N-glycosylation sites, and found several in regions very likely to affect protein folding and/or substrate binding (Fig. 1). We performed multi-site-directed mutagenesis on seven sites (Fig. 1; Table 1; Supplementary Figs. S2 and S3). Glycosylation recognition sequences were abolished by conservative substitutions, in most cases Asn → Gln.

### 3.2 Mutations at most N-glycosylation sites do not impair enzyme activity *in vitro*

**Fig. 2:**
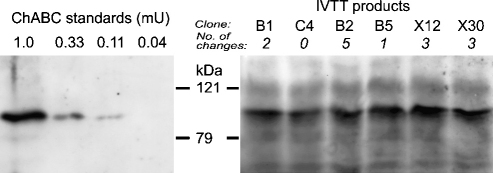
Western blot of products of the IVTT reactions detected with anti-chondroitinase antibody. *Left*, commercial chondroitinase ABC; *right*, IVTT products from the six clones indicated. The products comigrate with commercial chondroitinase ABC, and similar amounts are produced by all clones.

**Fig. 3:**
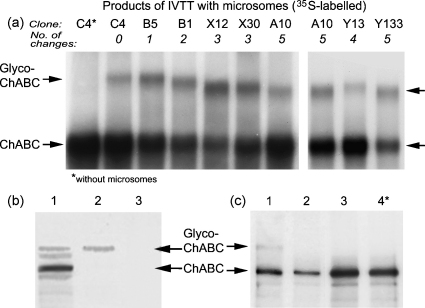
SDS-PAGE of IVTT reactions with microsomes to assess glycosylation. IVTT reactions were labeled with ^35^S-Met (a and c) or biotinylated lysine (b) and incubated with canine microsomes. After SDS-PAGE and blotting, the products were visualised. (a) Products of different clones. The lower arrow indicates unglycosylated chondroitinase ABC, which comigrated with commercial chondroitinase (not shown); the upper arrow indicates the upper (glycosylated) band, which was not produced without microsomes (lane 1). The glycosylated band migrates faster in products of clones with several mutations of N-glycosylation sites. (b) Confirmation that the upper band is protected from Proteinase K, indicating that it has been internalised in the microsomes (clone C4). Lane 1, IVTT with microsomes; Lane 2, the same plus Proteinase K; Lane 3, the same plus Triton X-100 to lyse the microsomes. (c) Confirmation that the upper band is digested by N-glycosidase (clone C4). Lane 1, IVTT with microsomes; Lanes 2 and 3, the same plus N-glycosidase (1 μg, 10 μg); Lane 4, IVTT without microsomes. (Lanes 1 and 2 are shown at higher contrast because they contained less material.)

The different constructs were tested for activity by transcription and translation in the rabbit reticulocyte lysate system (IVTT), and the products assayed by the colorimetric assay (Morgan–Elson reaction). All constructs produced similar amounts of protein. This was demonstrated either by Western blot using an antibody to the bacterial enzyme (Fig. 2), or by labeling the products with biotinylated lysine or ^35^S-methionine (Fig. 3a).

All the clones listed produced similar levels of activity in the Morgan–Elson reaction (data not shown). One mutation, Thr 340 to Ala, however abolished enzyme activity but the alternative mutation, Asn 338 to Gln, was well tolerated. Thus it is possible to make the required changes without compromising enzyme activity.

### 3.3 Mutations at N-glycosylation sites reduce glycosylation *in vitro*

Our hypothesis is that these sites are being inappropriately glycosylated in a eukaryotic system. To test this, and identify which of the sites are actually glycosylated, we compared different constructs in the reticulocyte lysate system in the presence of canine microsomes (endoplasmic reticulum preparation), which incorporate and N-glycosylate the nascent polypeptide as in an animal cell.

The unmodified enzyme (C4) was heavily glycosylated in the presence of microsomes (Fig. 3a, lane 2). The unglycosylated form produced in the absence of microsomes was seen as a band of around 110 kDa. In the presence of microsomes, an additional band was seen at higher Mr, as expected for glycosylation. To confirm that it was internalised by the microsomes, a Proteinase K protection experiment was performed (Fig. 3b). N-linked glycosylation occurs only within intact microsomes, and this assay makes use of the protection afforded the translocated protein domain by the microsomal membrane. Thus, translocated proteins are protected from exogenously added protease, and only the glycosylated form remains. To confirm that it was indeed glycosylated, samples were digested with N-glycosidase (Fig. 3c, lanes 2 and 3), and the high-Mr band disappeared as expected.

The glycosylated products of clones C4 and B5 comigrate (Fig. 3a), so the mutation of Asn-751 in B5 made no visible difference to the glycosylation. This indicates that Asn-751 is not detectably glycosylated, consistent with the sequence flanking Asn-751 which makes N-glycosylation unlikely (Petrescu et al., 2004). Conversely, the glycosylated products of clones with mutations at other sites show increased mobility on the gel (Fig. 3a). Comparison of these products with the mutations they contain (Table 1) indicates that Asn-675, 515, 345, 338 and 282 are all glycosylated as predicted. The glycosylation of Asn-515 is of particular significance because it lies in the cleft which constitutes the enzyme's active site.

Similar experiments with other constructs (not shown) have shown that Asn-856 and 963 are also glycosylated, but Asn-836 is not. Thus 7 out of 9 sites so far tested are glycosylated as predicted.

### 3.4 Chondroitinase ABC with reduced N-glycosylation is secreted in active form from transfected cells

**Fig. 4:**
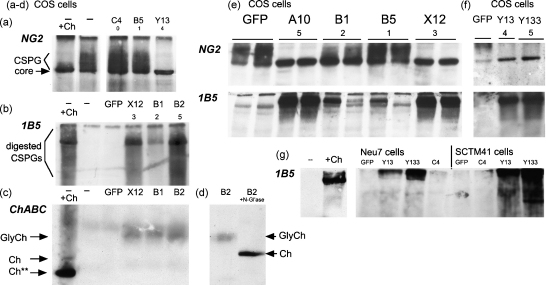
Chondroitinase ABC with reduced N-glycosylation is secreted in active form from transfected cells. Neu7 conditioned medium (a source of CSPGs) was placed on transfected cells for 24 h, then analysed by Western blotting, with antibodies against (a) the CSPG, NG2; (b) carbohydrate stubs produced by chondroitinase action (antibody 1B5); (c) chondroitinase ABC. Each lane is labeled according to the clone used for transfection, with the number of mutated glycosylation sites listed below. In (a, b, c and g), the first two lanes are positive and negative controls with Neu7 medium not exposed to transfected cells. Samples marked “+Ch’ase” were digested with commercial chondroitinase *in vitro* before SDS-PAGE. (a) Probed for NG2. Lanes 1 and 2, controls: in medium from untransfected cells, NG2 appears largely as a characteristic ‘smear’ as expected due to the GAG chains (lane 2), and this is all converted to core protein by digestion with commercial chondroitinase (lane 1). Lanes 3 and 4: No change in medium incubated with COS7 cells transfected with initial clone C4 or B5. Lane 5: With mutant Y13, chondroitinase activity eliminates the GAG chain smear and all NG2 appears as core protein. (b) Probed for 1B5 stub epitope. Neu7 conditioned medium, and the same incubated with GFP-transfected COS7 cells, shows little immunoreactivity (lanes 2 and 3), but digestion with commercial chondroitinase *in vitro* generates extensive reactivity (lane 1). Lanes 4–6: Medium incubated with COS7 cells transfected with chondroitinase mutants; all generate immunoreactivity, maximal for clone B2. (c) Same blot re-probed for chondroitinase ABC. Commercial chondroitinase (lane 1) shows both full-length band (Ch) and a shorter band (Ch**) due to proteolytic activity during incubation with medium. Clones X12, B1, and B2 all generate a diffuse chondroitinase band (GlyCh: partially glycosylated). (d) The same with a sample digested *in vitro* with N-glycosidase (lane 2). The diffuse band collapsed to a sharp band of the same Mr as commercial chondroitinase. (e and f) Blots of additional clones, probed for NG2 (e) and for 1B5 stub (f). These blots show a range of activity from clone B5 (little or no activity) to clone Y133 (the most active of all). (g) The same experiment with transfected Neu7 and SCTM41 cells, probed for 1B5 stub epitope. As the intensity of 1B5 staining varies between gels, the activity rankings in Table 1 are the consensus of several experiments in which subsets of clones were compared on single gels.

To test for secretion of active chondroitinase, we transfected each of the constructs into COS7 cells, and assayed conditioned medium by Western blotting for the effect of chondroitinase on CSPGs. These cells do not synthesise significant amounts of CSPGs, so as a source of CSPGs we incubated the cells after transfection with conditioned medium from the Neu7 cell line. These astrocyte-like cells (Fok-Seang et al., 1995) secrete substantial amounts of the axon-inhibitory CSPG, NG2, into the medium and the matrix (Smith-Thomas et al., 1994; Fidler et al., 1999). NG2 comprises a core protein of ∼290 kDa, and a proportion of the molecules are glycanated and therefore run as a higher-Mr smear on a gel (Levine and Nishiyama, 1996). Chondroitinase activity is demonstrated by reduction or loss of the glycanated smear, which is converted into the core protein. Therefore to assess chondroitinase activity, we applied Neu7 conditioned medium to the transfected COS7 cells from 24 h to 48 h post-transfection, then analysed the medium by Western blotting for NG2 (Fig. 4a).

In addition, blots were probed with the antibody 1B5, which recognises a carbohydrate ‘stub’ epitope that remains on the core proteins after chondroitinase has cleaved off the CSPG chains (Fig. 4b). This is a sensitive marker of chondroitinase activity as it detects the appearance of a novel product rather than the diminution of a substrate.

The Western blots (Fig. 4a, b, e and f) showed that the initial clone C4 (encoding unmodified bacterial chondroitinase) had no activity, but in contrast clone B1, with two N-glycosylation sites mutated, had significant activity as shown by both antibodies. Clones with three or more sites mutated had even more activity: they produced complete degradation of the NG2 GAG chains, and they produced levels of stub reactivity comparable with *in vitro* treatment with commercial chondroitinase ABC.

The same blots were re-probed with antibody against chondroitinase (Fig. 4c). A diffuse band was seen which represents partially glycosylated chondroitinase. Its identity was confirmed by N-glycosidase digestion *in vitro*, which converted it to a sharp band that comigrated with commercial chondroitinase (Fig. 4d). The initial clone C4 produced a variable amount of this band, or none, suggesting that glycosylation of the unmodified sequence interfered with secretion to a variable extent, but any protein produced was always inactive. Clones which produced chondroitinase activity in the medium always produced this chondroitinase band, and its mobility was slightly faster for clones that produced more activity, confirming that the additional mutations in those clones had further reduced the amount of N-glycosylation of the enzyme.

Additional clones are shown in Fig. 4e and f, including the clone with the highest activity obtained so far, clone Y133, which has mutations at 5 N-glycosylation sites. The results from all experiments are summarised in Table 1.

**Fig. 5:**
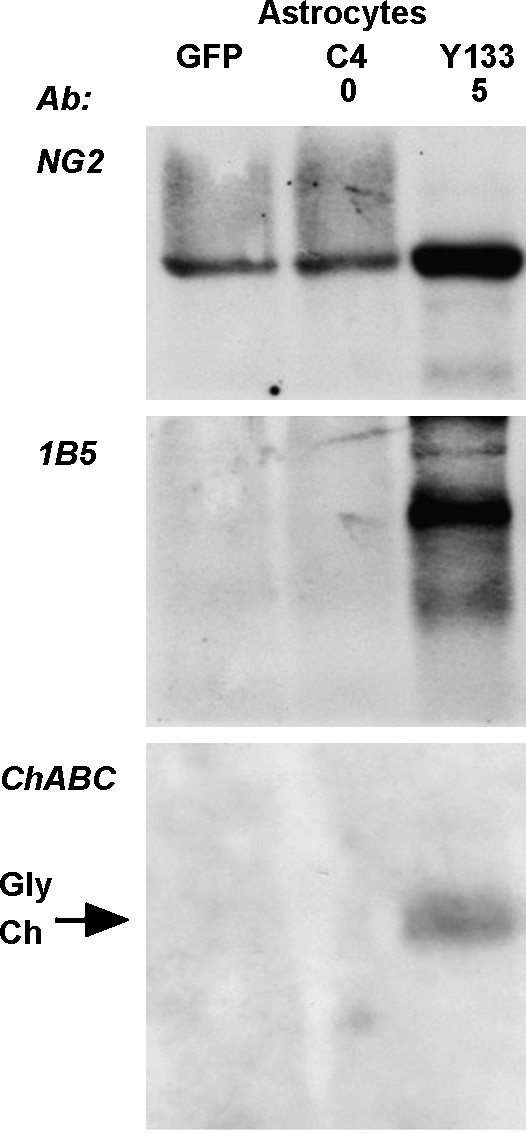
Chondroitinase ABC with reduced N-glycosylation is also secreted from primary astrocytes. The same experiment as in Fig. 4, with transfected primary astrocytes, probed for NG2, 1B5 stub epitope, and chondroitinase ABC. Again, clone C4 is inactive and clone Y133 is highly active.

To test for activity in cells of nervous system origin, we transfected two glial cell lines: SCTM41 cells derived from Schwann cells (Wilby et al., 1999), and Neu7 cells derived from astrocytes. Again, medium from confluent Neu7 cells was added as a source of CSPGs (since newly transfected Neu7 cells do not synthesise significant amounts of CSPGs for 48 h after transfection; data not shown). The results were similar to those with COS7 cells (Fig. 4g), confirming that the unmodified sequence C4 was inactive but modified sequences Y13 and especially Y133 were active. The same result was obtained with primary astrocytes from neonatal rat cortex (Fig. 5).

## 4 Discussion

In this study, we have shown that bacterial chondroitinase is inappropriately glycosylated by mammalian cells and that this has a detrimental effect on secretion of active enzyme, which can be overcome by removing strategic glycosylation sites.

The occurrence of N-glycosylation was demonstrated first using *in vitro* transcription–translation. In the presence of microsomes, chondroitinase synthesised from all our constructs was internalised (proteinase-protected) and N-glycosylated (slower-migrating). Clones with glycosylation site mutations produced faster-migrating bands, confirming that the glycosylation was reduced as intended.

Secretion of N-glycosylated chondroitinase by transfected COS7 cells was investigated by Western blots. The initial clone C4 (encoding the native bacterial sequence) produced either glycosylated chondroitinase or none. This stochastic behaviour could be expected if the block to secretion is self-amplifying, and thus sensitive to slight fluctuations in the initial impairment in the secretory pathway. The block might be initiated by exposure of hydrophobic sequences, or aggregation of misfolded protein, or inappropriate interaction with the secretory machinery. Any secreted chondroitinase from this clone lacked detectable enzymatic activity. Likewise clone B5, with only one mutation, produced secreted but inactive protein. Thus the secreted protein was in an inactive form, presumably due to carbohydrate chains either distorting the protein structure or producing steric hindrance of the active site. We conclude that the initial sequence allowed internalisation and glycosylation in the ER, but variably impeded transit through the secretory pathway, and invariably blocked secretion of active enzyme.

We then showed that ablation of strategic glycosylation sites resulted in secretion of active enzyme from transfected cells. Enzyme activity was demonstrated by two Western blot assays for degradation of CSPG GAG chains in the medium. As the experiment used pre-conditioned medium as the only source of CSPGs, this conclusively showed that the chondroitinase was secreted and acting extracellularly.

The improvement in secreted activity was attributable to the change in glycosylation, rather than to any changes in translation efficiency of the gene, given that the least-modified clones could secrete detectable but inactive enzyme (above), and the activity of the enzymes following transfection correlated with the number of glycosylation sites that had been ablated (Table 1). Clone B5, with only Asn-751 mutated, was still essentially inactive. Clone B1 (two sites mutated) showed some activity. Clones with three or more sites mutated all showed strong activity, comparable to that of commercial chondroitinase. The most active is clone Y133, which also produces the largest amount of chondroitinase immunoreactivity in conditioned medium.

It is likely that the effect is due to several specific sites, rather than merely the number of sites mutated. Asn-751 may be immaterial, as it is unlikely to be N-glycosylated at all according to the rules of Petrescu et al. (2004), and our *in vitro* assay showed that deleting this site had no effect on the mobility of the glycosylated protein (Fig. 4, clone B5). Conversely, Asn-515 seems to be important as it lies in the active site. We conclude that the mutations required for efficient secretion of active enzyme (yielding activity similar to the commercial enzyme) are those eliminating glycosylation at Asn-282, 336, 345, and 515, and that mutation of Asn-675 increases the activity still further.

It is important to identify these individual sites where N-glycosylation is deleterious. It is also important to test mutants *in vitro* to determine whether the enzyme activity has been compromised before they can be tested for secretability. For example a Thr-Ala substitution at Thr-338 abolished activity in the *in vitro* assay (data not shown), whereas a mutation at Asn-336, eliminating the same N-glycosylation site, was well tolerated. In addition, we have mutated other potential N-glycosylation sites and found that some changes actually prevent the secretion of the enzyme (unpublished data).

Cafferty et al. (2007) have reported production of *Proteus* chondroitinase ABC in transgenic mice, using the GFAP promoter and the original bacterial signal sequence to express it in astrocytes, and demonstrating activity in the injured CNS by staining with a stub antibody. However the stub antibody can detect small amounts of chondroitinase product, and indeed it could have detected products of intracellular chondroitinase activity on nascent CSPGs which had subsequently been secreted. Thus, efficient secretion of the enzyme was not proven. Moreover, the astrocytic expression was not sufficient to allow corticospinal axons to regenerate beyond the lesion, unlike the injection experiments of Bradbury et al. (2002), and neuronal expression was not attempted. As we have shown that mutations of glycosylation sites are required to achieve secretion of active enzyme in cultured cells, it is clearly important to use these mutants in future transgenic experiments for optimal expression.

Curinga et al. (2007) have recently reported expression of a different bacterial enzyme, chondroitinase AC, in mammalian cells, using an immunoglobulin signal sequence and adenovirus vector. However the specific activity of the secreted product was low.

This work extends our knowledge of the conditions required to express bacterial proteins in eukaryotic cells, highlighting one of the cryptic signals that may be recognised in bacterial sequences. This may now be applied to other situations where bacterial proteins need to be expressed by eukaryotic cells.

Bacterial chondroitinase ABC has shown considerable promise, a treatment for spinal cord injury. A gene expressing it in secretable form could be transfected into cells for grafting into the CNS injury site (e.g. Wilby et al., 1999). It could also be expressed from viral vectors to target it to neurons and/or glia in the injured CNS. These goals now appear achievable given that ablation of strategic glycosylation sites results in secretion of active enzyme.

## Figures and Tables

**Table 1:** Mutations of N-glycosylation sites.

Clone number:	C4	B5	B1	X12	X30	Y13	B2	Y133	A10	A106
Positions changed:	0	1	2	3	3	4	5	5	5	4
Asn-751	–	N-Q	N-Q	N-Q	N-Q	N-Q	N-Q	N-Q	N-Q	-
Asn-515 (S-A)	–		S-A	S-A	S-A	S-A	S-A	S-A	S-A	S-A
Asn-345	–			N-Q		N-Q	N-Q	N-Q	N-Q	N-Q
Asn-338	–								N-Q	N-Q
Asn-282	–				N-K	N-K	N-K	N-K	N-Q	N-Q
Asn-675	–							N-Q		
Asn-836	–						N-D			

Activity after transfection:	–	(+/−)	+	++	++	++	++	+++	++	++

The table indicates whether each clone had a mutation at each of seven Asn-X-(Ser/Thr) sites. Mutations were Asn-Gln (N-Q), Asn-Lys (N-K), Asn-Asp (N-D), or Ser-Ala (S-A; at position +2 relative to the Asn), as indicated.
