# Supplementary material for: Modification of N-glycosylation sites allows secretion of bacterial chondroitinase ABC from mammalian cells
Source: J Biotechnol. 2010 Jan 15;145(2):103–10. doi: 10.1016/j.jbiotec.2009.11.002 (PMC2809921; doi:10.1016/j.jbiotec.2009.11.002)
Supplement: Supplementary file 1 [file mmc1.doc]

E.M. Muir, I. Fyfe, S. Gardiner, L. Li, P. Warren, J.W. Fawcett*, R.J. Keynes, J.H. Rogers

**‘Modification of N-glycosylation sites allows secretion of bacterial chondroitinase ABC from mammalian cells’**

**On-line Supplementary Figures (S1 – S3)**

**Supplementary Figure S1:**

| *nt* |  |  |  |  |  |  |  | gcg | gcc | **gcc** | **atg** | **g**ag | gca | cga | gt**g** | **Kozak** |
| --- | --- | --- | --- | --- | --- | --- | --- | --- | --- | --- | --- | --- | --- | --- | --- | --- |
| *aa* |  |  |  |  |  |  |  |  |  |  | M | E | A | R | V | NotI, NcoI |
|  | gcc | tgg | gga | gcg | ctg | gcc | gga | cct | ctg | cgg | gtt | ctc | tgc | gtc | ctg |  |
|  | A | W | G | A | L | A | G | P | L | R | V | L | C | V | L |  |
| 270 | tgc | tgc | ctg | ttg | ggc | cgc | gcc | atc | gcc | **g**cc | ac**t** | ag**t** | aat | cct | gca |  |
| 16 | C | C | L | L | G | R | A | I | A | A | T | S | N | P | A |  |
| 315 | ttt | gat | cct | aaa | aat | ctg | atg | cag | tca | gaa | att | tac | cat | ttt | gca |  |
| 31 | F | D | P | K | N | L | M | Q | S | E | I | Y | H | F | A |  |
| 360 | caa | aat | aac | cca | tta | gca | gac | ttc | tca | tca | gat | aaa | aac | tca | ata |  |
| 46 | Q | N | N | P | L | A | D | F | S | S | D | K | N | S | I |  |
| 405 | cta | acg | tta | tct | gat | aaa | cgt | agc | att | atg | gga | aac | caa | tct | ctt |  |
| 61 | L | T | L | S | D | K | R | S | I | M | G | N | Q | S | L |  |
| 450 | tta | tgg | aaa | tgg | aaa | ggt | ggt | agt | agc | ttt | act | tta | cat | aaa | aaa |  |
| 76 | L | W | K | W | K | G | G | S | S | F | T | L | H | K | K |  |
| 495 | ctg | att | gtc | ccc | acc | gat | aaa | gaa | gca | tct | aaa | gca | tgg | gga | cgc |  |
| 91 | L | I | V | P | T | D | K | E | A | S | K | A | W | G | R |  |
| 540 | tca | tct | acc | ccc | gtt | ttc | tca | ttt | tgg | ctt | tac | aat | gaa | aaa | ccg |  |
| 106 | S | S | T | P | V | F | S | F | W | L | Y | N | E | K | P |  |
| 585 | att | gat | ggt | tat | ctt | act | atc | gat | ttc | gga | gaa | aaa | ctc | att | tca |  |
| 121 | I | D | G | Y | L | T | I | D | F | G | E | K | L | I | S |  |
| 630 | acc | agt | gag | gct | cag | gca | ggc | ttt | aaa | gta | aaa | tta | gat | ttc | act |  |
| 136 | T | S | E | A | Q | A | G | F | K | V | K | L | D | F | T |  |
| 675 | ggc | tgg | cgt | gct | gtg | gga | gtc | tct | tta | aat | aac | gat | ctt | gaa | aat |  |
| 151 | G | W | R | A | V | G | V | S | L | N | N | D | L | E | N |  |
| 720 | cga | gag | atg | acc | tta | aat | gca | acc | aat | acc | tcc | tct | gat | ggt | act |  |
| 166 | R | E | M | T | L | N | A | T | N | T | S | S | D | G | T |  |
| 765 | caa | gac | agc | att | ggg | cgt | tct | tta | ggt | gct | aaa | gtc | gat | agt | att |  |
| 181 | Q | D | S | I | G | R | S | L | G | A | K | V | D | S | I |  |
| 810 | cgt | ttt | aaa | gcg | cct | tct | aat | gtg | agt | cag | ggt | gaa | atc | tat | atc |  |
| 196 | R | F | K | A | P | S | N | V | S | Q | G | E | I | Y | I |  |
| 855 | gac | cgt | att | atg | ttt | tct | gtc | gat | gat | gct | cgc | tac | caa | tgg | tct |  |
| 211 | D | R | I | M | F | S | V | D | D | A | R | Y | Q | W | S |  |
| 900 | gat | tat | caa | gta | aaa | act | cgc | tta | tca | gaa | cct | gaa | att | caa | ttt |  |
| 226 | D | Y | Q | V | K | T | R | L | S | E | P | E | I | Q | F |  |
| 945 | cac | aac | gta | aag | cca | caa | cta | cct | gta | aca | cct | gaa | aat | tta | gcg |  |
| 241 | H | N | V | K | P | Q | L | P | V | T | P | E | N | L | A |  |
| 990 | gcc | att | gat | ctt | att | cgc | caa | cgt | cta | att | aat | gaa | ttt | gtc | gga |  |
| 256 | A | I | D | L | I | R | Q | R | L | I | N | E | F | V | G |  |
| 1035 | ggt | gaa | aaa | gag | aca | aac | ctc | gca | tta | gaa | gag | aat | atc | agc | aaa | G1 |
| 271 | G | E | K | E | T | N | L | A | L | E | E | N | I | S | K |  |
| 1080 | tta | aaa | agt | gat | ttc | gat | gct | ctt | aat | att | cac | act | tta | gca | aat |  |
| 286 | L | K | S | D | F | D | A | L | N | I | H | T | L | A | N |  |
| 1125 | ggt | gga | acg | caa | ggc | aga | cat | ctg | atc | act | gat | aaa | caa | atc | att |  |
| 301 | G | G | T | Q | G | R | H | L | I | T | D | K | Q | I | I |  |
| 1170 | att | tat | caa | cca | gag | aat | ctt | aac | tcc | caa | gat | aaa | caa | cta | ttt |  |
| 316 | I | Y | Q | P | E | N | L | N | S | Q | D | K | Q | L | F |  |
| 1215 | gat | aat | tat | gtt | att | tta | ggt | aat | tac | acg | aca | tta | atg | ttt | aat | G2 |
| 331 | D | N | Y | V | I | L | G | N | Y | T | T | L | M | F | N |  |
| 1260 | att | agc | cgt | gct | tat | gtg | ctg | gaa | aaa | gat | ccc | aca | caa | aag | gcg |  |
| 346 | I | S | R | A | Y | V | L | E | K | D | P | T | Q | K | A |  |
| 1305 | caa | cta | aag | cag | atg | tac | tta | tta | atg | aca | aag | cat | tta | tta | gat |  |
| 361 | Q | L | K | Q | M | Y | L | L | M | T | K | H | L | L | D |  |
| 1350 | caa | ggc | ttt | gtt | aaa | ggg | agt | gct | tta | gtg | aca | acc | cat | cac | tgg |  |
| 376 | Q | G | F | V | K | G | S | A | L | V | T | T | H | H | W |  |
| 1395 | gga | tac | agt | tct | cgt | tgg | tgg | tat | att | tcc | acg | tta | tta | atg | tct |  |
| 391 | G | Y | S | S | R | W | W | Y | I | S | T | L | L | M | S |  |
| 1440 | gat | gca | cta | aaa | gaa | gcg | aac | cta | caa | act | caa | gtt | tat | gat | tca |  |
| 406 | D | A | L | K | E | A | N | L | Q | T | Q | V | Y | D | S |  |
| 1485 | tta | ctg | tgg | tat | tca | cgt | gag | ttt | aaa | agt | agt | ttt | gat | atg | aaa |  |
| 421 | L | L | W | Y | S | R | E | F | K | S | S | F | D | M | K |  |
| 1530 | gta | agt | gct | gat | agc | tct | gat | cta | gat | tat | ttc | aat | acc | tta | tct |  |
| 436 | V | S | A | D | S | S | D | L | D | Y | F | N | T | L | S |  |
| 1575 | cgc | caa | cat | tta | gcc | tt**g** | **c**t**g** | tta | ct**g** | gag | cct | gat | gat | caa | aag |  |
| 451 | R | Q | H | L | A | L | L | L | L | E | P | D | D | Q | K |  |
| 1620 | cgt | atc | aac | tta | gtt | aat | act | ttc | agc | cat | tat | atc | act | ggc | gca |  |
| 466 | R | I | N | L | V | N | T | F | S | H | Y | I | T | G | A |  |
| 1665 | tt**g** | acg | caa | gtg | cca | ccg | ggt | ggt | aaa | gat | ggt | tta | cgc | cct | gat |  |
| 481 | L | T | Q | V | P | P | G | G | K | D | G | L | R | P | D |  |

| 1710 | ggt | aca | gca | tgg | cga | cat | gaa | ggc | aac | tat | ccg | ggc | tac | tct | ttc |  |
| --- | --- | --- | --- | --- | --- | --- | --- | --- | --- | --- | --- | --- | --- | --- | --- | --- |
| 495 | G | T | A | W | R | H | E | G | N | Y | P | G | Y | S | F | Frameshift |
| 1755 | cca | gcc | ttt | aaa | aat | gcc | tct | cag | ctt | att | tat | tta | tta | cgc | gat | G3 |
| 511 | P | A | F | K | N | A | S | Q | L | I | Y | L | L | R | D |  |
| 1800 | aca | cca | ttt | tca | gtg | ggt | gaa | agt | ggt | tgg | aat | aac | ctg | aaa | aaa |  |
| 526 | T | P | F | S | V | G | E | S | G | W | N | N | L | K | K |  |
| 1845 | gcg | atg | gtt | tca | gcg | tgg | atc | tac | agt | aat | cca | gaa | gtt | gga | tta |  |
| 541 | A | M | V | S | A | W | I | Y | S | N | P | E | V | G | L |  |
| 1890 | ccg | ctt | gca | gga | aga | cac | cct | ttt | aac | tca | cct | tcg | tta | aaa | tca |  |
| 556 | P | L | A | G | R | H | P | F | N | S | P | S | L | K | S |  |
| 1935 | gtc | gct | caa | ggc | tat | tac | tgg | ctt | gcc | atg | tct | gca | aaa | tca | tcg |  |
| 571 | V | A | Q | G | Y | Y | W | L | A | M | S | A | K | S | S |  |
| 1980 | cct | gat | aaa | aca | ctt | gca | tct | att | tat | ctt | gcg | att | agt | gat | aaa |  |
| 586 | P | D | K | T | L | A | S | I | Y | L | A | I | S | D | K |  |
| 2025 | aca | caa | aat | gaa | tca | act | gct | att | ttt | gga | gaa | act | att | aca | cca |  |
| 601 | T | Q | N | E | S | T | A | I | F | G | E | T | I | T | P |  |
| 2070 | gcg | tct | tta | cct | caa | ggt | ttc | tat | gcc | ttt | aat | ggc | ggt | gct | ttt |  |
| 616 | A | S | L | P | Q | G | F | Y | A | F | N | G | G | A | F |  |
| 2115 | ggt | att | cat | cgt | tgg | caa | gat | aaa | atg | gtg | aca | ctg | aaa | gct | tat |  |
| 631 | G | I | H | R | W | Q | D | K | M | V | T | L | K | A | Y |  |
| 2160 | aac | acc | aat | gtt | tgg | tca | tct | gaa | att | tat | aac | aaa | gat | aac | cgt |  |
| 646 | N | T | N | V | W | S | S | E | I | Y | N | K | D | N | R |  |
| 2205 | tat | ggc | cgt | tac | caa | agt | cat | ggt | gtc | gct | caa | ata | gtg | agt | aat | G5 |
| 661 | Y | G | R | Y | Q | S | H | G | V | A | Q | I | V | S | N |  |
| 2250 | ggc | tcg | cag | ctt | tca | cag | ggc | tat | cag | caa | gaa | ggt | tgg | gat | tgg |  |
| 676 | G | S | Q | L | S | Q | G | Y | Q | Q | E | G | W | D | W |  |
| 2295 | aat | aga | atg | caa | ggg | gca | acc | act | att | cac | ctt | cct | ctt | aaa | gac |  |
| 690 | N | R | M | Q | G | A | T | T | I | H | L | P | L | K | D |  |
| 2340 | tta | gac | agt | cct | aaa | cct | cat | acc | tta | atg | caa | cgt | gga | gag | cgt |  |
| 706 | L | D | S | P | K | P | H | T | L | M | Q | R | G | E | R |  |
| 2385 | gga | ttt | agc | gga | aca | tca | tcc | ctt | gaa | ggt | caa | tat | ggc | atg | atg |  |
| 721 | G | F | S | G | T | S | S | L | E | G | Q | Y | G | M | M |  |
| 2430 | gca | ttc | gat | ctt | att | tat | ccc | gcc | aat | ct**g** | gag | cgt | ttt | gat | cct | G4 |
| 736 | A | F | D | L | I | Y | P | A | N | L | E | R | F | D | P |  |
| 2475 | aa**c** | ttc | act | gcg | aaa | aag | agt | gta | tta | gcc | gct | gat | aat | cac | tta |
| 751 | N | F | T | A | K | K | S | V | L | A | A | D | N | H | L |
| 2520 | att | ttt | att | ggt | agc | aat | ata | aat | agt | agt | gat | aaa | aat | aaa | aat |  |
| 766 | I | F | I | G | S | N | I | N | S | S | D | K | N | K | N |  |
| 2565 | gtt | gaa | acg | acc | tta | ttc | caa | cat | gcc | att | act | cca | aca | tta | aat |  |
| 781 | V | E | T | T | L | F | Q | H | A | I | T | P | T | L | N |  |
| 2610 | acc | ctt | tgg | att | aat | gga | caa | aag | ata | gaa | aac | atg | cct | tat | caa |  |
| 796 | T | L | W | I | N | G | Q | K | I | E | N | M | P | Y | Q |  |
| 2655 | aca | aca | ctt | caa | caa | ggt | gat | tgg | tta | att | gat | agc | aat | ggc | aat |  |
| 811 | T | T | L | Q | Q | G | D | W | L | I | D | S | N | G | N |  |
| 2700 | ggt | tac | tta | att | act | caa | gca | gaa | aaa | gta | aat | gta | agt | cgc | caa |  |
| 826 | G | Y | L | I | T | Q | A | E | K | V | N | V | S | R | Q |  |
| 2745 | cat | cag | gtt | tca | gcg | gaa | aat | aaa | aat | cgc | caa | ccg | aca | gaa | gga |  |
| 841 | H | Q | V | S | A | E | N | K | N | R | Q | P | T | E | G |  |
| 2790 | aac | ttt | agc | tcg | gca | tgg | atc | gat | cac | agc | act | cgc | ccc | aaa | gat |  |
| 856 | N | F | S | S | A | W | I | D | H | S | T | R | P | K | D |  |
| 2835 | gcc | agt | tat | gag | tat | atg | gtc | ttt | tta | gat | gcg | aca | cct | gaa | aaa |  |
| 871 | A | S | Y | E | Y | M | V | F | L | D | A | T | P | E | K |  |
| 2880 | atg | gga | gag | atg | gca | caa | aaa | ttc | cgt | gaa | aat | aat | ggg | tta | tat |  |
| 886 | M | G | E | M | A | Q | K | F | R | E | N | N | G | L | Y |  |
| 2925 | cag | gtt | ctt | cgt | aag | gat | aaa | gac | gtt | cat | att | att | ctc | gat | aaa |  |
| 901 | Q | V | L | R | K | D | K | D | V | H | I | I | L | D | K |  |
| 2970 | ctc | agc | aat | gta | acg | gga | tat | gcc | ttt | tat | cag | cca | gca | tca | att |  |
| 916 | L | S | N | V | T | G | Y | A | F | Y | Q | P | A | S | I |  |
| 3015 | gaa | gac | aaa | tgg | atc | aaa | aag | gtt | aat | aaa | cct | gca | att | gtg | atg |  |
| 931 | E | D | K | W | I | K | K | V | N | K | P | A | I | V | M |  |
| 3060 | act | cat | cga | caa | aaa | gac | act | ctt | att | gtc | agt | gca | gtt | aca | cct |  |
| 946 | T | H | R | Q | K | D | T | L | I | V | S | A | V | T | P |  |
| 3105 | gat | tta | aat | atg | act | cgc | caa | aaa | gca | gca | act | cct | gtc | acc | atc |  |
| 961 | D | L | N | M | T | R | Q | K | A | A | T | P | V | T | I |  |
| 3150 | aat | gtc | acg | att | aat | ggc | aaa | tgg | caa | tct | gct | gat | aaa | aat | agt |  |
| 976 | N | V | T | I | N | G | K | W | Q | S | A | D | K | N | S |  |
| 3195 | gaa | gtg | aaa | tat | cag | gtt | tct | ggt | gat | aac | act | gaa | ctg | acg | ttt |  |
| 991 | E | V | K | Y | Q | V | S | G | D | N | T | E | L | T | F |  |

| 3240 | acg | agt | tac | ttt | ggt | att | cca | caa | gaa | atc | aaa | ctc | tcg | cca | ctc |  |
| --- | --- | --- | --- | --- | --- | --- | --- | --- | --- | --- | --- | --- | --- | --- | --- | --- |
| 1006 | T | S | Y | F | G | I | P | Q | E | I | K | L | S | P | L |  |
| 3285 | cct | tga |  |  |  |  |  |  |  |  |  |  |  |  |  |  |
| 1021 | P | * |  |  |  |  |  |  |  |  |  |  |  |  |  |  |

**Supplementary Figure S1:**

**Sequence of initial chondroitinase ABC gene (clone C4).**

This is the gene sequence which we used as the basis for expression and mutation studies, encoding the secreted *P. vulgaris* chondroitinase ABC sequence as in Ryan et al.(1994) and Prabhakar et al.(2005a), with a signal sequence from mouse MMP-2. Numbering of nucleotides and codons in the chondroitinase coding region (nucl. 297 onwards) is as in Sato et al.(1994); the section that was frame-shifted in Sato et al. (1994) is marked. The sequence up to nucl. 297 is replaced by a NotI site (underlined: used for cloning into pcDNA3.1) overlapping a Kozak initiation sequence (bold, purple), then the coding sequence for mouse MMP-2 signal sequence, which is joined to nucl. 297 of the chondroitinase ABC sequence. (Nucls 297-300, and the encoded Ala-Ala sequence, comprise identical residues representing the cleavage site of the signal sequence in both MMP-2 and chondroitinase ABC.) From there onwards the sequence is identical to the *P. vulgaris* chondroitinase ABC gene, except for individual nucleotides in bold where synonymous substitutions have been made to alter restriction sites or to substitute codons more frequently used in mammalian genes. Underlined sections labelled G1 to G5 indicate matches to oligonucleotides used to introduce mutations in N-glycosylation sites [Suppl. Fig. S2].

**Supplementary Figure S2.**

G1(b)

Start nt 1052, End nt 1099

N282 → Q

| 5’ |  |  |  |  |  |  |  |  |  |  |  |  |  |  |  | 3’ |  |
| --- | --- | --- | --- | --- | --- | --- | --- | --- | --- | --- | --- | --- | --- | --- | --- | --- | --- |
| c | ctc | gca | tta | gaa | gag | CaG | atc | agc | aaa | tta | aaa | agt | gat | ttc | gat | gc |  |
|  | L | A | L | E | E | Q | I | S | K | L | K | S | D | F | D |  |  |

G2(e)

Start nt 1224, End nt 1280

N338 → Q, N345 → Q, L342 codon improved.

| 5’ |  |  |  |  |  |  |  |  |  |  |  |  |  |  |  |  |  | 3’ |  |
| --- | --- | --- | --- | --- | --- | --- | --- | --- | --- | --- | --- | --- | --- | --- | --- | --- | --- | --- | --- |
| gtt | att | tta | ggt | CaG | tac | acg | aca | Cta | atg | ttt | CaG | att | agc | cgt | gct | tat | gtg | ctg |  |
| V | I | L | G | Q | Y | T | T | L | M | F | Q | I | S | R | A | Y | V | L |  |

G3

Start nt 1756, End nt 1804

(N515) S517 → A, L519 and L522 codons improved.

| 5’ |  |  |  |  |  |  | PvuII | |  |  |  |  |  |  |  | 3’ |  |
| --- | --- | --- | --- | --- | --- | --- | --- | --- | --- | --- | --- | --- | --- | --- | --- | --- | --- |
| ca | gcc | ttt | aaa | aat | gcc | Gct | cag | ctG | att | tat | CtG | tta | cgc | gat | aca | cc |  |
|  | A | F | K | N | A | A | Q | L | I | Y | L | L | R | D | T |  |  |

G4

Start nt 2446, End nt 2486

L745 restored, N751 → Q

| 5’ |  |  |  | XhoI | |  |  |  |  |  |  |  | 3’ |  |
| --- | --- | --- | --- | --- | --- | --- | --- | --- | --- | --- | --- | --- | --- | --- |
| at | ccc | gcc | aat | cTC | gag | cgt | ttt | gat | cct | CaG | ttc | act | gcg |  |
|  | P | A | N | L | E | R | F | D | P | Q | F | T | A |  |

G5(b)

Start nt 2237, End nt 2277

N675 → Q

| 5’ |  |  |  |  |  |  | PvuII | |  |  |  |  |  | 3’ |  |
| --- | --- | --- | --- | --- | --- | --- | --- | --- | --- | --- | --- | --- | --- | --- | --- |
| a | ata | gtg | agt | CaG | ggc | gcg | cag | ctG | tca | cag | ggc | tat | cag | c |  |
|  | I | V | S | Q | G | S | Q | L | S | Q | G | Y | Q |  |  |

**Supplementary Figure S2.**

**Sequences of primers used to introduce mutations.**

Introduced mutations are in red capital letters.

**Supplementary Figure S3.**


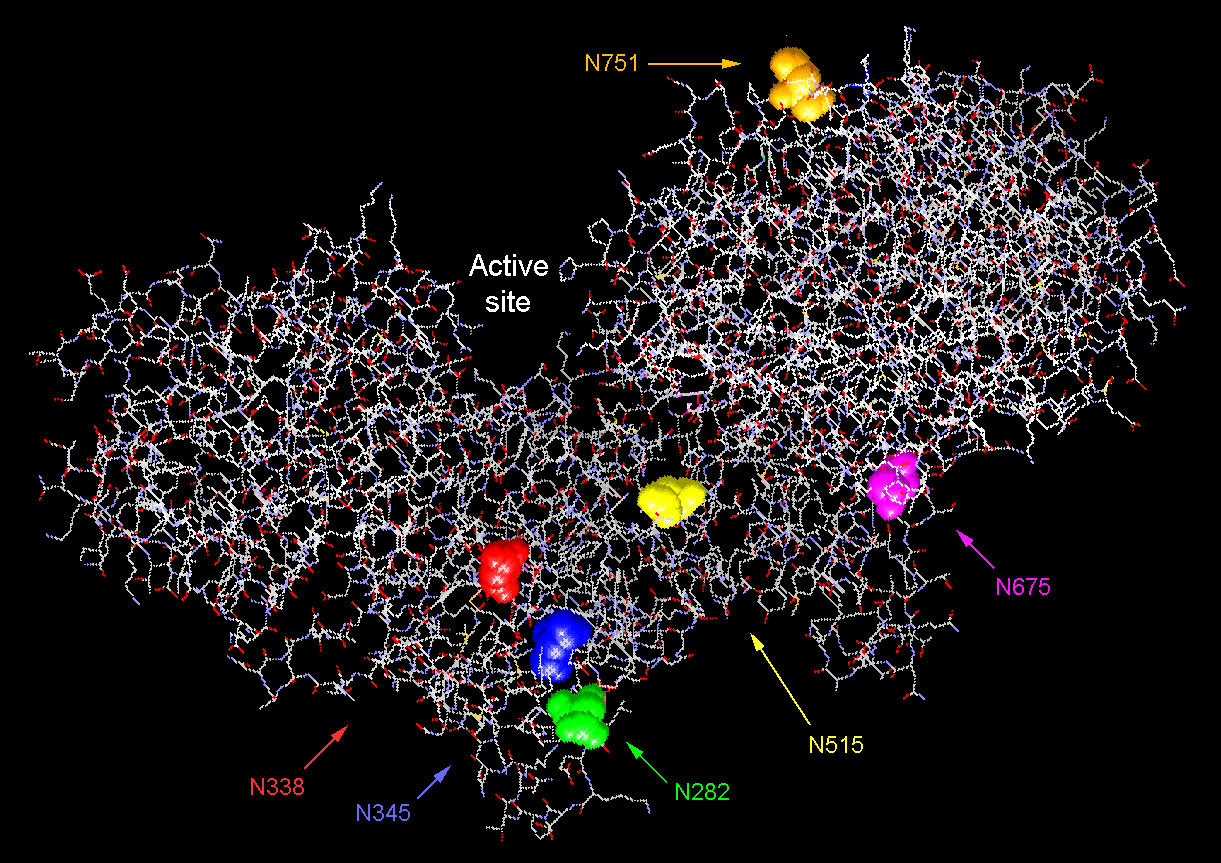


**Supplementary Figure S3. 3D structure of bacterial chondroitinase ABC, showing putative glycosylation sites that have been eliminated (colour version of Fig.1).** The structure is from Huang et al. (2003). The active site is on the right-hand flank of the cleft (Prabhakar et al., 2005b). Six potential N-glycosylation sites predicted to affect enzyme structure or activity are highlighted. Different constructs differ as follows (see Table 1): B5 v C4, Asn 751; B1 v B5, Asn 515; X12 v B1, Asn 345; X30 v B1, Asn 282; and Y13 v A10, Asn 338. (A seventh site, Asn-836, was mutated in one clone but is not glycosylated.)
